# Efficacy and safety of cryoablation for localized renal tumor as an alternative approach to partial nephrectomy

**DOI:** 10.3389/fonc.2023.1235705

**Published:** 2023-10-03

**Authors:** Irène Barjolle, Loic Ah-Thiane, Eric Frampas, Georges Karam, Jérôme Rigaud, Arthur David

**Affiliations:** ^1^ Department of Radiology, University Hospital (CHU) Nantes, Nantes University, Nantes, France; ^2^ Department of Radiotherapy, Western Cancer institute (ICO) René Gauducheau 44805 St-Herblain, Nantes University, Nantes, France; ^3^ Department of Urology, University Hospital (CHU) Nantes, Nantes University, Nantes, France

**Keywords:** cryoablation, interventional radiology, localized renal cancer, partial nephrectomy, renal tumor, T1b renal tumor

## Abstract

**Introduction:**

Renal cryoablation displays a profile of high tolerance, including in a frail population. Cryoablation appears as a validated alternative treatment to surgery for renal tumors smaller than 4 cm. However, evidence is lacking for larger tumors, despite encouraging data for tumors up to 7 cm.

**Material and methods:**

This retrospective descriptive study of a population with a stage T1b renal tumor treated by cryoablation was conducted at the Nantes University Hospital between January 2009 and July 2021. Primary endpoint was 3-year rate of local recurrence. Secondary endpoints included technical efficacy, overall and cancer-specific survivals, and safety assessment.

**Results:**

A total of 63 patients were analyzed. Three-year rate of local recurrence was 11.1%. Primary and secondary technical efficacies were achieved in 88.9% and 96.8% of patients, respectively, and 3-year overall and cancer-specific survival were 87.3% and 95.2%, respectively. Most patients (73%) experienced no complications, 13% of patients had minor (CIRSE grades 1 or 2) adverse effects, and 13% had severe but non-lethal (CIRSE grade 3) adverse effects. One patient died following cryoablation due to colic perforation. The most common AE (all grades) was hemorrhage (9.5%).

**Discussion:**

This study showed a good efficacy and safety of cryoablation for renal tumors up to 7 cm (T1b). Our results were consistent with a rather sparse literature and contributed to guide future recommendations about cryoablation as an alternative to surgery for T1b renal tumors.

## 1 Introduction

The main treatment of localized renal cell carcinoma (RCC) used to be limited to surgery (1), with either radical or partial nephrectomy (2). Such interventions come with immediate surgical or anesthetic risks, and long-term sequelae. Among them, loss of renal function can be important following radical nephrectomy, but not neglectable after partial nephrectomy (PN) (3–5). Minimally invasive approaches have been developed in interventional radiology, including percutaneous radiofrequency ablation (RFA), cryoablation (CA), or microwave ablation (MWA), which presented some benefits in limiting blood loss and post-procedure pain, shortening operating time and stay length, and better preserving renal function compared to surgical approach (6). Since percutaneous ablation showed comparable oncological outcomes to surgery for small RCC (7–9), both the American Urological Association (AUA) and the European Association of Urology (EAU) have validated RFA and CA as possible treatments for RCC inferior to 4 cm (T1a) (10, 11). For larger tumors up to 7 cm (T1b), nephrectomy remains, nonetheless, the standard reference. As a matter of fact, RFA is technically limited to small masses, as its efficacy rapidly decreases with tumor size (12), and CA, despite being technically feasible for larger tumors, currently lacks of evidence to be recommended (13). However, CA showed encouraging results in this setting and could deserve further investigations (14). We hypothesized that CA could be indicated for larger renal tumors, where surgical resection is currently the only validated treatment, thus offering an option for inoperable patients. The purpose of the present article was to assess the efficacy and safety of CA for treating patients with T1b RCC, as an alternative approach to surgery.

## 2 Materials and methods

### 2.1 Study design and participants

We retrospectively collected data from all patients treated with CA for local RCC in our university hospital in France. Data collection was based on medical records available at the time of data collection (during first trimester 2022). Patients were included if they were treated by CA between January 2009 and July 2021 for a localized primitive or unique recurrence of a renal tumor T1b (41–70 mm). All patients treated during this period were eligible, thanks to the per-procedure images archived in our picture archiving and communication system. Tumor size was measured directly on pre-procedure imaging and could be somewhat subjective, particularly in the absence of injected imaging, which could render the measurement imprecise. Post-procedural complications were recorded in the patient file and graded according to their severity using the CIRSE classification (15), which may have been somewhat subjective. Patients were excluded if the follow-up was inferior to 12 months. Patients gave written consent to use their data for research purposes and publication.

### 2.2 Treatment modality

**Figure 1:**
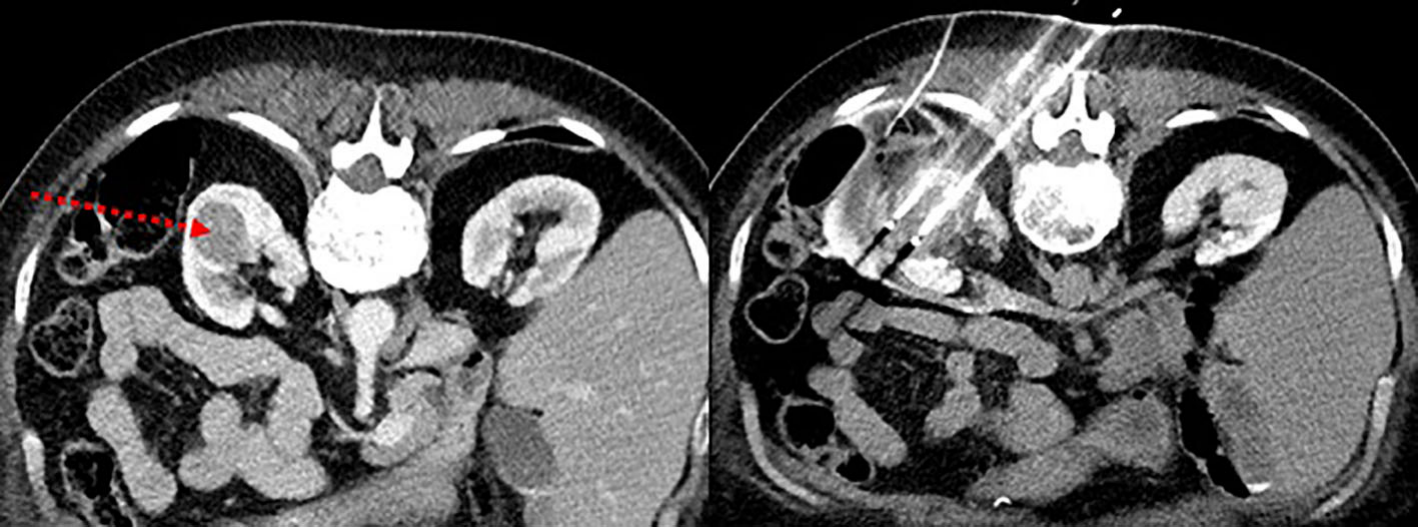
Pre- and per-procedure images of renal cryoablation. (Left picture) CT scan showing a left renal mass of 42 mm to treat (see arrow). (Right picture) Visualization of the inserted cryoprobes and the ice ball clearly circumscribing the tumor lesion.

**Figure 2:**
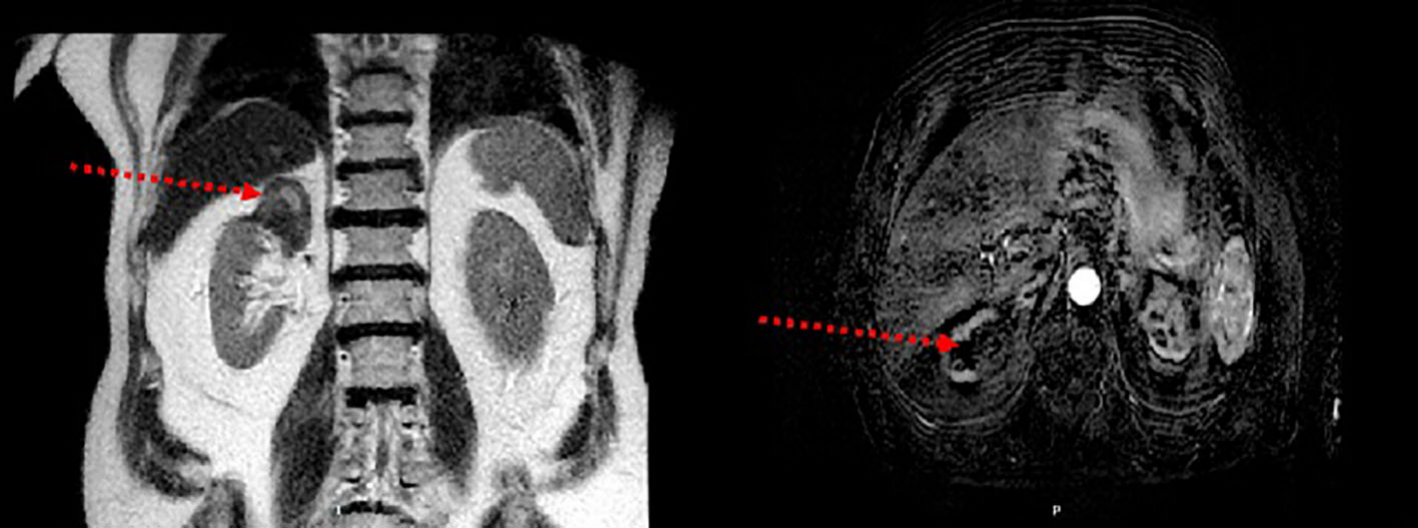
Post-procedure magnetic resonance imaging. (Left picture) The right upper polar cryoablation zone (see arrow) is in discreetly heterogeneous T2 hyposignal. (Right picture) T1 sequence injected at subtracted arterial time showing no nodular enhancement over the cryoablation area (see arrow).

The CA procedure was performed under general anesthesia. Prior to CA, tumoral selective embolization was allowed in case of major bleeding risk. The tumor to treat was first located on contrast-enhanced CT scan. The type and number of cryoprobes were selected according to the size and shape of the tumor. The median number of needles used per tumor treated was four. The cryoprobes were inserted with a maximum spacing of 15 mm. Once the probes were correctly positioned under CT control, a complete CA cycle was performed including two freezing phases of 10 min each separated by a thawing phase, passive for 9 min and active using helium or electricity for 1 min. The extension of the formed ice ball, the good tumor coverage, and the proximity to the adjacent organs were monitored under CT. Treatment was considered complete when the ice ball extended at least 5 mm beyond the tumor margins in all planes. In case of proximity to vulnerable tissues (intestine, ureter, and pancreas, for example), a protective technique by hydrodissection (with saline or glucose, possibly opacified with iodinated contrast medium) was used. At the end of a CA cycle, active thawing was used to facilitate removal of cryoprobes. Follow-up MR imaging was performed at 3, 6, 12, 24, and 36 months. In case of tumor residue, a second CA was performed, with a new evaluation 3 months after. **Figures 1** and **2** illustrates the CA procedure through examples of imaging (CT scan and MRI) before, during, and after the process.

### 2.3 Outcomes and statistical analysis

The primary outcome was 3-year rate of local recurrence (LR). LR was defined as a relapse in the site treated by CA on follow-up imaging after an initial complete response.

Secondary outcomes were primary and secondary technical efficacies (TE), 3-year overall survival (OS), distant metastasis (DM), cancer-specific survival (CSS), and safety. Primary TE was defined as no residue identified on imaging within 3 months following a first CA. Secondary TE was defined as no residue identified on imaging within 3 months following the second salvage CA. OS was defined as the time from inclusion to death from any cause. DM was defined as occurrence of secondary lesions elsewhere than the kidneys. CSS was defined as the time from inclusion to death from renal cancer. Complications were assessed according to CIRSE classification (15).

Qualitative data were expressed as proportion or percentage and quantitative data as median and interquartile range. OS and CSS were analyzed using the Kaplan–Meier method.

## 3 Results

### 3.1 Patients baseline characteristics

From January 2009 to July 2021, 63 patients were included in the analysis. Baseline characteristics are presented in **Table 1**. Only one of the patients in the population was a carrier of a genetic anomaly with renal risk (tuberous sclerosis of Bourneville); 16% of the population had a single anatomical or functional kidney. Two patients had a tumoral selective embolization prior to CA. Furthermore, five patients previously received a local treatment by either percutaneous ablation or partial nephrectomy.

**Table 1:** Patients’ characteristics.

Median age in years (IQR)	79.5 (70.9–83.0)
Sex (female)	11/63 (17.5%)
Median body mass index (IQR)	27 (24–31)
Genetic anomaly	1/63 (1.6%)
Single kidney	10/63 (15.9%)
ASA score	
1	2/63 (3.2%)
2	21/63 (33.3%)
3	36/63 (57.1%)
4	3/63 (4.8%)
NA	2/63 (3.2%)

### 3.2 Tumors baseline characteristics

**Table 2** summarizes the characteristics of the tumors treated by CA. Most tumors were clear cell carcinomas (89%). The size of treated tumors ranged from 41 and 60 mm, with a median size of 45 mm.

**Table 2:** Tumors’ characteristics.

Median size in mm (IQR)	45 (42–48.5)
Right-sided tumor	34/63 (53.9%)
Histology	
Clear cells	56/63 (88.9%)
Papillary	5/63 (7.9%)
Chromophobe	2/63 (3.2%)
ISUP grade	
1	15/63 (23.8%)
2	17/63 (27%)
3	6/63 (9.5%)
4	0/63 (0%)
NA	25/63 (39.7%)
Localisation	
Exophytic	26/63 (41.3%)
Parenchymal	24/63 (38.1%)
Central	13/63 (20.6%)
RENAL score	
6	1/63 (1.6%)
7	8/63 (12.7%)
8	15/63 (23.8%)
9	18/63 (28.6%)
10	14/63 (22.2%)
11	7/63 (11.1%)

### 3.3 Efficacy

**Figure 3:**
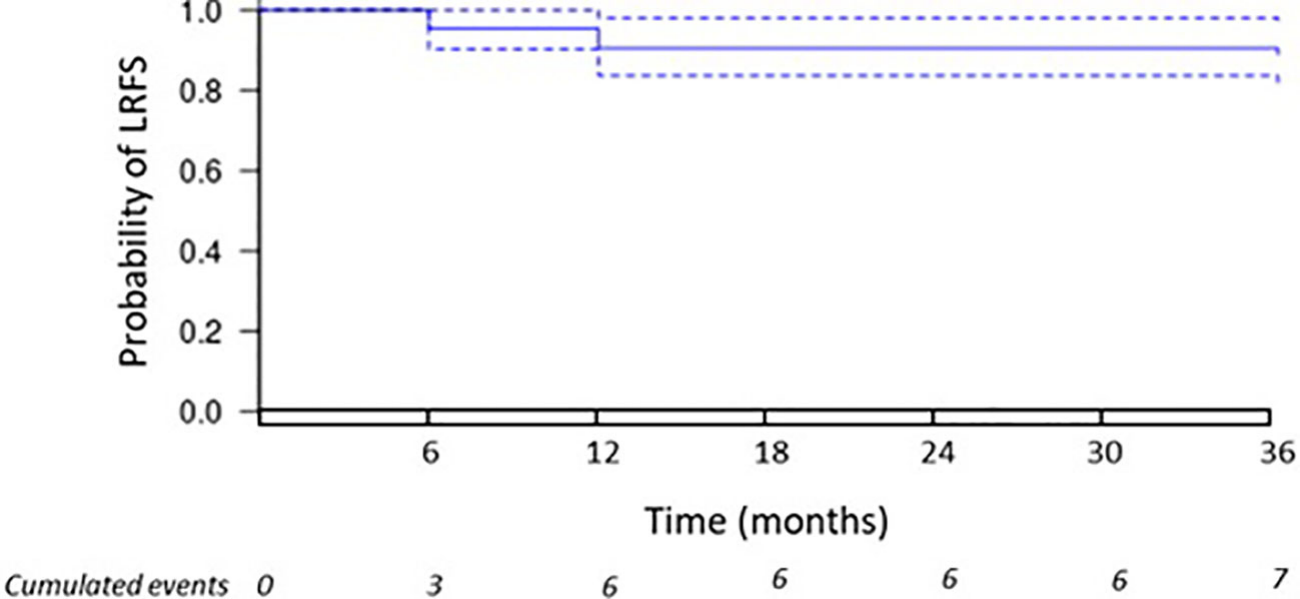
Local recurrence. The Kaplan–Meier curve displays the estimate of local recurrence-free survival (LRFS).

As primary outcome, 3-year rate of LR was 11.1%. Indeed, out of the 63 patients, 7 had a recurrence in the treated site. For the record, three patients relapsed at 6 months, three patients at 12 months, and one patient at 36 months. **Figure 3** displays the Kaplan–Meier curves estimating the survival without local recurrence.

**Figure 4:**
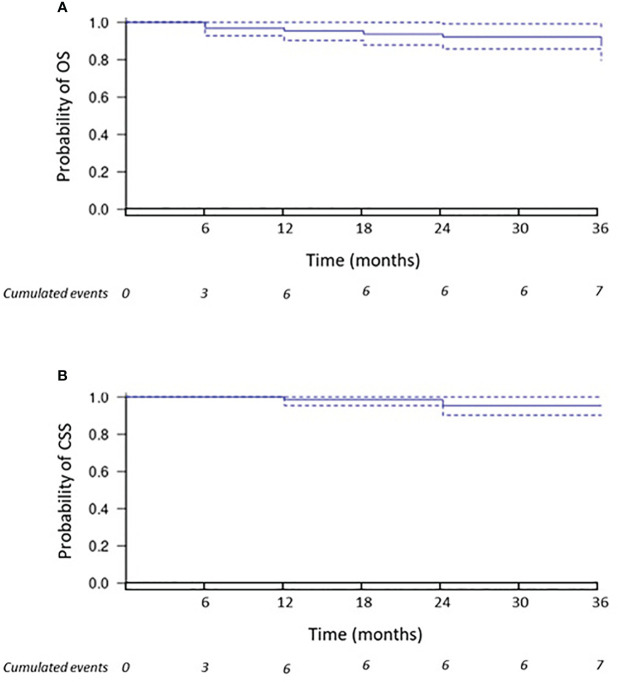
Overall survival and cancer-specific survival. The Kaplan–Meier curve **(A)** displays the estimate of overall survival (OS). The Kaplan–Meier curve **(B)** displays the estimate of cancer-specific survival (CSS).

Furthermore, primary and secondary TE were achieved in 56/63 patients (88.9%) and in 61/63 (96.8%), respectively. In addition, 3-year OS and CSS were 87.3% and 95.2%, respectively. Indeed, eight patients died: three from cancer progression, one from CA procedure, four from other causes unrelated to neither renal cancer nor CA. **Figures 4A, B** display the Kaplan–Meier curves estimating the OS and CSS, respectively. DM occurred in 7/63 (11.1%) patients having a metastatic progression in lungs and/or bones.

### 3.4 Safety

Adverse effects (AEs) are reported in **Table 3**. The vast majority of patients (46/63 patients, 73%) experienced no complications. Eight of the 63 patients (13%) had minor (CIRSE grades 1 or 2) AE, and 8/63 patients (13%) had severe but non-lethal (CIRSE grade 3) AE. No complications causing long-term sequelae (CIRSE grades 4 or 5) were reported. However, one patient presented a CIRSE grade 6 AE: a colic perforation that led to death. The most common AE (all grades) was hemorrhage (9.5%), with, for instance, pleural clotting evacuated by thoracotomy and intercostal or perirenal hematoma. In addition, 4.8% presented a pneumothorax, treated by exsufflation or draining. Other complications included infections (hypothermia, obstructive pyelonephritis, and abdominal abscess).

**Table 3:** Adverse effects following cryoablation, according to CIRSE classification.

Complications (CIRSE grade)	17/63 (27%)
None	46/63 (73%)
1	5/63 (7.9%)
2	3/63 (4.8%)
3	8/63 (12.7%)
4	0/63 (0%)
5	0/63 (0%)
6	1/63 (1.6%)
Types of complications	
Hemorrhagic	6/63 (9.5%)
Infectious	3/63 (4.8%)
Vascular	1/63 (1.6%)
Pneumothorax	3/63 (4.8%)
Other benign	3/63 (4.8%)
Death	1/63 (1.6%)

## 4 Discussion

Our study showed that CA had very promising oncological outcomes in terms of local control, TE, metastasis control, CSS, and OS at 3 years. In our study, the initial technical success rate (absence of visible tumor residue on the first check at 3 months) was 88.9%, with a secondary success rate (after a new procedure) of 96.8%.The local recurrence rate was 11.3% after an average follow-up of 29 months; the rate of progression to metastatic disease was 11% at the end of follow-up. A recent systematic review and meta-analysis by Cazalas et al. included six retrospective studies, which included between 23 and 48 patients each, for a total of 204 patients treated by CA for a T1b RCC, with a mean follow-up ranging between 14 and 72 months (16). By pooling these studies, the rates of LR, primary and secondary TE, and distant metastasis occurrence were 10.1% (19/188), 91.9% (137/149), 96.2% (76/79), and 6.1% (7/115), respectively. Our results were consistent with these findings (8, 17–21). In addition, a retrospective study found that larger T1 tumors could benefit from CA as much as T1a tumors, since there was no statistical difference in local recurrence between tumors <3 cm and >3 cm (p =0.15) (22). A study even suggested the feasibility of CA for T3a RCC for inoperable patients (23).

Our study showed a good tolerance of CA, with a majority of patients having no complications. The rates of AE that we reported were very similar to those in the meta-analysis by Cazalas et al., with 12.5% and 9.6% of patients experiencing minor and major AE, respectively (16). The most common AE after CA was hemorrhage, whereas pneumothorax or infections were possible but less frequent, in agreement with previous studies (24–26). In order to limit hemorrhage, two of our patients at high risk of bleeding underwent selective transarterial embolization prior to CA and had no hemorrhagic complication, representing a possible option (27). To note, one patient died from a direct complication of CA, namely, a cryolesion-induced colonic perforation. This constitutes one of the limitations of CA when the tumor location is unfavorable, even if techniques such as hydrodissection aim to reduce the risk. As a result, CA must be discussed by a multidisciplinary team to ensure that it is carried out under the right conditions, with a favorable benefit/risk balance.

Despite satisfying efficacy and safety, CA does not currently appear as a validated alternative to nephrectomy in neither AUA nor EAU guidelines, and its place is still a matter of debate for T1b RCC (10, 11). An analysis of 448 procedures reported a good preservation of renal function with CA and with PN (28), while a cohort study of 118 patients treated by either CA or PN did not find any difference in LR (p =0.7), DM (p =0.2), or CSS (p =0.8) between the two groups (29). Another comparative study did not find any difference in CSS (p =0.5) or OS (p =0.15), but highlighted a significantly higher rate of LR with CA compared to PN (p =0.019) (30). The AblatT1b study-UroCCR 80 also found that thermal ablation techniques (CA and MWA) led to a significantly higher rate of LR (14.6% vs. 4%; p =0.02) but lower rates of major AE (5.3% vs. 0%; p <0.001) (31). A meta-analysis by Uhlig et al. showed that CA was associated with higher rate of LR (incidence rate ratio =4.13, p <0.05) and with higher all-cause mortality (incidence rate ratio =2.58, p <0.001) compared to PN, but without any difference in CSS (32). Another meta-analysis by Yanagisawa confirmed that PN was associated with lower rate of LR (risk ratio =0.41, 95%CI [0.23–0.75]), but without any difference in CSS or DM (33). However, it should be reminded the difficulties to interpret the results due to the retrospective nature of the studies and the unmatched cohorts. In fact, outcomes could be biased, given that patients treated by CA are often inoperable with more comorbidities. For instance, a retrospective study emphasized that patients treated by CA rather than PN were significantly older (odds ratio = 11.4, 95%CI [3.33–45.1]) (34).

Our study has several limitations. First, it was a retrospective analysis with the bias inherent to its nature (e.g., bias in patients selection and follow-up). Second, it included a limited number of patients; mainly because CA is not a treatment supported by a high level of evidence for T1b RCC. Third, it was a non-comparative study, without direct comparison to surgery or to other minimally invasive techniques in interventional radiology, knowing there are some differences between them (35, 36). Nevertheless, this work is one of the largest single-centered data gathering on the subject and provides valuable pieces of information to support the use of CA for T1b RCC, consistent with the existing literature. The criteria to select accurately patients benefiting the most from CA remain to be determined through larger-scale prospective studies. This study could precede a prospective cohort comparison with the reference treatment, which remains partial or total nephrectomy in this indication and could suggest a less invasive interventional approach in selected patients with T1b renal tumors. Future practical applications could focus on percutaneous thermoablation techniques for patients with T1 renal tumors and a single kidney (37), or for fully endophytic tumors, where CA provided comparable results to PN (38).

## 5 Conclusion

CA is a minimally invasive technique in interventional radiology that is recommended for T1a RCC, as a validated alternative to surgical nephrectomy. On the contrary, it is not recommended for T1b RCC, in which case PN remains the standard reference. However, for inoperable patients, CA appears to be appealing, since some encouraging results were published. However, the existing literature is rather sparse, and this work contributed to bring some valuable information supporting the use of CA for T1b RCC.

## Data availability statement

The datasets that support the findings of the current study are not publicly available. Each request for access to the dataset will be granted upon reasonable request sent to the corresponding author. The corresponding author declares that he had full access to all the data in the study and takes responsibility for the integrity of the data and the accuracy of the data analysis.

## Ethics statement

The requirement of ethical approval was waived by Comité d’éthique CHU de Nantes for the studies involving humans because Generic consent of non-opposition to research. The studies were conducted in accordance with the local legislation and institutional requirements. The ethics committee/institutional review board also waived the requirement of written informed consent for participation from the participants or the participants’ legal guardians/next of kin because Generic consent of non-opposition to research.

## Author contributions

IB: conceptualization, writing—original draft. LA-T: methodology, writing—original draft. EF: writing—review and editing. GK: writing—review and editing. JR: writing—review and editing. AD: writing—review and editing, conceptualization, and supervision. All the authors have read and approved the submitted manuscript.
